# Assessment of the Risk of Depression in Residents Staying at Long-Term Care Institutions in Poland During the COVID-19 Pandemic Depending on the Quality of Cognitive Functioning

**DOI:** 10.3389/fpsyg.2021.766675

**Published:** 2022-01-03

**Authors:** Michał Górski, Marta Buczkowska, Mateusz Grajek, Jagoda Garbicz, Beata Całyniuk, Kamila Paciorek, Aleksandra Głuszek, Renata Polaniak

**Affiliations:** ^1^Doctoral School of the Medical University of Silesia in Katowice, Department of Human Nutrition, Faculty of Health Sciences in Bytom, Medical University of Silesia in Katowice, Katowice, Poland; ^2^Department of Toxicology and Health Protection in the Occupational Environment, Faculty of Health Sciences in Bytom, Medical University of Silesia in Katowice, Katowice, Poland; ^3^Department of Public Health, Faculty of Health Sciences in Bytom, Medical University of Silesia in Katowice, Katowice, Poland; ^4^Doctoral School of the Medical University of Silesia in Katowice, Faculty of Health Sciences in Bytom, Medical University of Silesia in Katowice, Katowice, Poland; ^5^Department of Human Nutrition, Faculty of Health Sciences in Bytom, Medical University of Silesia in Katowice, Katowice, Poland

**Keywords:** COVID-19, SARS-CoV-2, elderly individuals, depression, dementia, long-term care institutions

## Abstract

**Background:** The development of the COVID-19 pandemic has prompted the implementation of many procedures to safeguard against further increases in illness. Unfortunately, this has drastically reduced residents’ contact with their families, which has increased feelings of loneliness and isolation. This is particularly difficult in long-term care facilities, where the risk of developing depression is higher than in the general population.

**Objectives:** The aim of the study was to assess the risk of depression among the residents of long-term care institutions in Poland during the COVID-19 pandemic and to determine the relationship between the risk of depression and the occurrence of cognitive impairment in the study group.

**Methods:** The study included 273 residents from long-term care institutions in Poland. The risk of depression was determined based on an originally designed questionnaire. The cognitive state of the subjects was assessed using the screening test Mini-Mental State Examination (MMSE). Both the depression risk assessment and the MMSE test were conducted twice: in March and December 2020.

**Results:** In March, severe dementia was present in 28.2% of the residents and normal MMSE scores were observed in 16.1% of the subjects; in December, the prevalence of severe dementia increased to 31.1% and that of normal scores decreased to 10.3%. In March, no participant was found to be at high risk of depression and moderate risk was observed in 14.3% of the subjects; in December, 2.6% of the residents had a high risk score and 45.4% had a moderate risk score. Statistical analysis revealed that higher MMSE scores correspond with a higher risk of depression.

**Conclusion:** A higher risk of depression was observed with the development of the pandemic. Residents with cognitive impairment were characterised by a lower risk of depression compared to individuals with normal MMSE scores. During the study, progression of cognitive impairment was observed in the residents.

## Introduction

In 2019 in Wuhan in China, a number of cases of unexplained pneumonia occurred. In January 2020, the Chinese Center For Disease Control and Prevention (CCDC) detected a novel coronavirus in a patient’s throat sample (Zhu et al., 2020). The World Health Organization (WHO) named the virus 2019-nCoV (“2019 novel coronavirus”). The name was subsequently changed to “severe acute respiratory syndrome coronavirus 2” (SARS-CoV-2) and the disease caused by the virus was named “coronavirus disease” (COVID-19) (World Health Organization [WHO], 2021). The SARS-CoV-2 virus soon spread to the whole world (Zhu et al., 2020).

In order to limit the spread of the virus, social distancing and the use of personal protective equipment were recommended. The authorities of different countries reacted in various ways, which involved the application of various measures such as closing schools, businesses and other public meeting places, cancelling public events or introducing restrictions in public transport (McKenzie and Adams, 2020). The procedures applied and fear of the spreading pathogen have had a significant impact on the everyday life of whole populations.

Particular attention should be paid to the influence of the pandemic on people’s mental health. Scientific research confirms an increase in stress levels, and rate of anxiety disorders and depression, which correlates with the COVID-19 pandemic (Babicki and Mastalerz-Migas, 2020; Luo et al., 2020; Smith et al., 2020). This is associated with both fear of contracting the disease and loneliness caused by isolation from the loved ones. The need for social bonding is deeply rooted in the human psyche, and isolation and loss of social relations can result in deteriorated cognitive function and decreased mood (Santini et al., 2020).

Observations revealed that the risk of acute symptoms and death associated with COVID-19 is higher in elderly individuals and patients with chronic comorbidities such as hypertension, heart disease or cancer, which are also more common in the elderly (Violant-Holz et al., 2020). Furthermore, a causal relationship is noted between COVID-19 transfection and the development of dementing disorders, including Alzheimer’s disease (Ciaccio et al., 2021). There is some evidence for an association of APOE ε4 (particularly ε4ε4 homozygote) with increased susceptibility to infectious disease and degeneration (Kloske and Wilcock, 2020; Ciaccio et al., 2021). It is suspected that SARS-CoV-2 infection may be a factor promoting neurodegeneration in individuals with susceptible genetic variants. Gal-3 and IL-1 proteins are also highly implicated, with increased levels of these proteins observed in patients with severe COVID-19. It has been hypothesised that increased levels of Gal-3 and IL-1 in patients with COVID-19 may also be involved in the damage leading to the development of Alzheimer’s disease (Cauchois et al., 2020; Tao et al., 2020; Ciaccio et al., 2021). Therefore, this age group is at a particularly high risk of mental disorders associated with fear of contracting COVID-19 and, at the same time, social isolation, which increases a sense of loneliness (Armitage and Nellums, 2020; Goethals et al., 2020). In addition, limitations on meeting people and recommendations to stay home lead to decreased levels of physical activity in older people, which also contributes to mental state deterioration (Goethals et al., 2020). Research confirms that social support of elderly individuals is associated with improvement in their mental health (Smith et al., 2020).

Depression is one of the most common mental diseases. The number of cases of depression is constantly increasing, particularly in the elderly (Bukhari et al., 2016; Uwabor et al., 2019). It is estimated that 20% of the world’s population of individuals aged over 65 suffer from depression; in this age group, depression may be additionally associated with neuropsychological disorders, cardiovascular diseases, diabetes or other conditions (Vilagut et al., 2016; Wilkinson and Izmeth, 2016). In the ICD-10 classification in force in Poland, the diagnostic criteria for depression include basic and additional symptoms which make it possible to diagnose and characterise the severity of depression. Basic symptoms include: lowered mood, loss of interests, decreased energy and increased tiredness. Additional symptoms include: low self-esteem and guilt, suicidal thoughts/behaviour, cognitive impairment, changes in psychomotor activity, sleep disturbances, changes in appetite associated with weight change. The diagnosis of depression requires two conditions:

(a) the symptom criterion (the patient must have at least two of the three core symptoms and additional symptoms, bringing the total number of symptoms to at least four);
(b) time criterion (episode lasts at least 2 weeks) (Domańska, 2018).

The residents of long-term care institutions such as care homes (Polish: *Domy Pomocy Społecznej*), and care and medical treatment centres (Polish: *Zakłady Opiekuńczo-Lecznicze*) are at a particularly high risk of mental problems during the COVID-19 pandemic. In many such facilities special precautions were introduced for the duration of the pandemic such as limitation of or ban on family visits, restricting contacts between the residents, and sometimes even between the residents and staff. Many institutions ceased running group activities for the residents and holding communal meals. Unfortunately, research shows that individuals who stay at care centres are more commonly affected by depression compared to individuals of similar age who are not under the care of such institutions (Guimarães et al., 2019; Knyszyńska et al., 2019; Tesky et al., 2019). In addition, an increase in the level of depression and anxiety during the COVID-19 pandemic among the residents of various long-term care institutions was observed (El Haj et al., 2020; Górski et al., 2020).

Some of the main health problems among elderly individuals, which are considered to be among the most significant geriatric problems, are cognitive impairment and dementia (Han et al., 2017). These disorders develop as a result of progressing neurodegenerative changes which lead to irreversible brain damage. They are characterised by progressing memory impairment, compromised ability to think, count and write, impaired orientation in time and place, and problems with communication and language (Jia et al., 2020). Cognitive impairment and dementia are often accompanied by emotional disturbances and affective diseases (Han et al., 2017; El Haj et al., 2020). Patients’ general activity levels systematically decline and they lose their independence in daily activities. The risk of cognitive impairment increases with age; during their lifetime, 10% of people worldwide are affected. It is estimated that in 2020, approximately 50 million people suffered from neurodegenerative disorders (Jia et al., 2020). These disorders are diagnosed in approximately 3% of individuals aged between 65 and 74, in approximately 20% of people aged between 75 and 84 and in as many as nearly 50% of individuals aged over 85 (Han et al., 2017; Mazurek et al., 2018; Jia et al., 2020).

The aim of the study was to assess the risk of depression among the residents of long-term care institutions in Poland at the beginning (March 2020) and during (December 2020) the COVID-19 pandemic and to determine the relationship between the risk of depression and the occurrence of cognitive impairment in the study group.

## Materials and Methods

### Study Group

The study included residents from long-term care institutions (care and medical treatment centres and care homes) located in Poland. The directors of each institution agreed for their centre to participate in the study.

The study was conducted in two stages: at the beginning of the pandemic, in March 2020, and after 9 months, in December 2020. The first stage of the study (March 2020) included 348 participants. Ultimately, the results of 273 participants who took part in both stages of the study were analysed. The difference between the first period of the study and the final number of subjects was due to refusal to participate in the second assessment in December or death of some of the respondents. Participation in the study was voluntary. The subjects whose cognitive functioning allowed them to familiarise themselves with the aim of the study expressed their independent and informed consent to participate in the study. For residents diagnosed with cognitive impairment double consent was required: that expressed by the resident themselves and that provided by their legal guardian/close family. Conducting the study did not require the authors to obtain approval from a bioethics committee in light of the Physician and Dentist Profession Act of December 5, 1996, which provides a definition of medical experimentation.

The main criteria for inclusion in the study were resident status in a given long-term care institution (the resident was supposed to have been staying in the centre for longer than 30 days), consent to participation in the study and maintenance of basic daily activity. Individuals who stayed at an institution for less than 30 days were excluded from the study (it was believed that conducting the study among newcomers could provide unreliable results due to ongoing process of adaptation to new conditions and environment), severely ill residents whose condition required prolonged bed rest (terminal stage of cancer, consequences of traffic accidents, disorders of consciousness, acute infectious diseases, e.g., pneumonia, COVID-19). Individuals with depression, treated for depression and those with acute positive symptoms associated with schizophrenia were also excluded from the study.

### Research Tool

The risk of depression was determined using an originally designed questionnaire which included 16 questions assessing the functioning of the residents and their mood stability, and questions regarding the residents’ demographics. The questionnaire was given the name: “Depressive Symptom Inventory”. The questionnaire had been previously validated. Validation was performed on 41 participants. They were asked to complete the “Depressive Symptom Inventory” twice with a 5-day interval in the study. The obtained results were statistically analysed to assess the reliability of the developed questionnaires. The internal consistency of the scales was examined by means of the α-Cronbach’s coefficient and by determining the correlation coefficients between the answers to individual questions and the total scale scores. Test-retest reliability was determined by comparing the results obtained when the same person completed the same questionnaire twice at a 5-day interval and by determining the intraclass correlation coefficient (ICC). A significant high correlation was found between the scores obtained for each question and the total score (*p* < 0.05, *r* > 0.64 in each case). The calculated α-Cronbach’s coefficient was high at 0.89, indicating very good internal consistency of the questionnaire.

A reliability analysis of the questionnaire was performed on the basis of questionnaires that were completed correctly twice. The level of reproducibility was determined using the ICC, which was 0.78. No statistically significant differences were found between the total scores and the scores for individual questions obtained after completing twice (on day 0 and day 5) (*p* > 0.05 in each case). Correlation coefficients were determined between answers to individual questions obtained during the first and second completion of the questionnaire. Significant high correlation was found between the results obtained for each question in case of double questioning (*p* < 0.05 and *r* > 0.51 in each case).

Prior to the present study, a pre-test was conducted to determine the cut-off values for each depression risk range. This was done using the PQStat software, where an intersection plot analysis of sensitivity and specificity was performed (in the present study, sensitivity = 0.85, specificity = 0.96). The form was filled in by psychologists following psychological assessment and observation of the resident. The questionnaire included questions regarding increased weepiness, decline in activity levels, depressed mood, concern about the future, increased helplessness, fear of something negative happening, more frequent inquiries about family members, worrying > 1 h/day, increased drowsiness (> 12 h/day), reluctance to leave one’s room, subjective sense of compromised cognitive functioning and thinking, emotional dysregulation, dysphoria, suicidal thoughts, suicidal behaviour, hope for rapid improvement of the situation. The possible answers were affirmative (“yes”) or negative (“no”). Questions 1–15 were rated on a 0–1 scale; an affirmative answer scored 1 point and a negative answer scored 0 points. In question 16, an affirmative answer resulted in deducting 1 point, and a negative answer involved adding 1 point. The total number of points scored was calculated (16 points was the maximum score), and the results were interpreted according to the scale below:

• Low risk of depression: 1–4 points;
• Moderate risk of depression: 5–9 points;
• High risk of depression: > 10 points.

All participants with dementia had a medical diagnosis (based on CT scan, MRI, medical history, symptoms, and psychological testing). The results of these tests were not included in the study because it is not related to the topic of the study. Level of dementia was assessed using the screening test Mini-Mental State Examination (MMSE). MMSE scores were adjusted for age and number of years of education. The scores were interpreted according to the standard presented below:

• Normal score: 30–27 points;
• Cognitive impairment without dementia: 26–24 points;
• Mild dementia: 23–19 points;
• Moderate dementia: 18–11 points;
• Severe dementia: 10–0 points.

Both the depression risk assessment and the MMSE test were conducted twice: in March and December 2020.

### Statistical Analysis

The current study presents detailed characteristics of the subjects with regard to gender, duration of stay at an institution, degree of dementia and risk of depression. The Shapiro–Wilk test was used to assess the normality of data distributions. The χ^2^ test, Kruskal-Wallis test and Wilcoxon test were used to test for significance of differences. Spearman’s non-parametric correlation test was used to analyse the relationships between the variables.

The results for which *p* < 0.05 were considered statistically significant. Statistical analysis was performed using Statistica 13.3 PL (StatSoft Polska, Kraków, Poland).

## Results

### Characteristics of the Study Group

The study included 273 residents, 51.6% (*N* = 141) of whom were women and 48.4% (*N* = 132) were men. Mean age was 80.81 ± 8.17 years. The largest age groups were 71–80-year-olds (*N* = 106; 38.8%) and 81–90-year-olds (*N* = 96; 35.2%), while individuals aged ≤ 60 years (*N* = 4; 1.5%) and > 100 (*N* = 2; 0.7%) belonged to the smallest age groups in the study. The duration of stay at the institutions differed between the subjects. At the beginning of the study (March 2020), the largest number of residents stayed at an institution for more than 24 months (*N* = 87; 31.9%), while the smallest number of participants spent 7–12 months there (*N* = 48; 17.6%). In March, severe dementia was present in 28.2% of the residents (*N* = 77), and a normal MMSE score was observed in 16.1% of the subjects (*N* = 44), while in December, the prevalence of severe dementia increased to 31.1% (*N* = 85) and that of normal scores decreased to 10.3% (*N* = 28). In March, no participant was found to be at high risk of depression, and moderate risk was observed in 14.3% of the subjects (*N* = 39), whereas in December, 2.6% of the residents (*N* = 7) had a high risk score and 45.4% had a moderate risk score (*N* = 124). No statistical differences in terms of gender were found for any of the investigated parameters. Detailed characteristics of the study group with regard to gender are presented in Table 1.

**TABLE 1:** Characteristics of the study group with regard to gender.

Variable	Total N;%	Gender		*p*-value*
		Women N;% 141; 51,6%	Men N;% 132; 48,4%	
**Duration of stay at the institution until the start of the study (months)**				
≤6	73; 26,7%	39;27,7%	34; 25,8%	
7–12	48; 17,6%	28; 19,8%	20; 15,1%	
13–24	65; 23,8%	35; 24,8%	30; 22,7%	0,44*
>24	87; 31,9%	39; 27,7%	48; 36,4%	
Presence of dementia (MMSE**) – 03.2020				
Normal score	44; 16,1%	21; 14,9%	23; 17,4%	
Cognitive impairment without dementia	29; 10,6%	17; 12,0%	12; 9,1%	
Mild dementia	72; 26,4%	40; 28,4%	32; 24,3%	0,43*
Moderate dementia	51; 18,7%	21; 14,9%	30; 22,7%	
Severe dementia	77; 28,2%	42; 29,8%	35; 26,5%	
Presence of dementia (MMSE**) – 12.2020				
Normal score	28; 10,3%	16; 11,4%	142; 9,1%	
Cognitive impairment without dementia	30; 11,0%	14; 9,9%	16; 12,1%	
Mild dementia	61; 22,3%	36; 25,5%	25; 18,9%	0,32*
Moderate dementia	69; 25,3%	29; 20,6%	40; 30,3%	
Severe dementia	85; 31,1%	46; 32,6%	39; 29,6%	
**Risk of depression – 03.2020**				
Low	234; 85,7%	123; 87,2%	111; 84,1%	
Moderate	39; 14,3%	18; 12,8%	21; 15,9%	0,45*
High	0; 0%	0; 0%	0; 0%	
**Risk of depression – 12.2020**				
Low	142; 52,0%	71; 50,4%	71; 53,8%	
Moderate	124; 45,4%	66; 46,8%	58; 43,9%	0,83*
High	7; 2,6%	4; 2,8%	3; 2,3%	

* χ^2^ test. **MMSE, Mini-Mental State Examination.

### Statistical Analysis

Statistical analysis revealed no relationship between the duration of stay at an institution and the presence of dementia and risk of depression.

**FIGURE 1:**
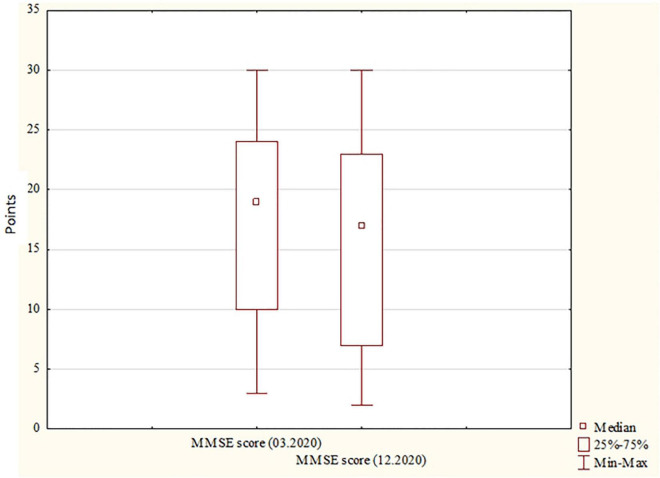
MMSE scores for March and December. MMSE, Mini-Mental State Examination. MMSE scores, the number of scores obtained from the MMSE.

Significant differences were found between the MMSE scores obtained in March and in December (*p* < 0.0001). The median score in March was higher than the one in December, which was reflected in a higher rate of moderate and severe dementia, with mild dementia becoming less frequent. MMSE scores for March and December are presented in Figure 1.

**FIGURE 2:**
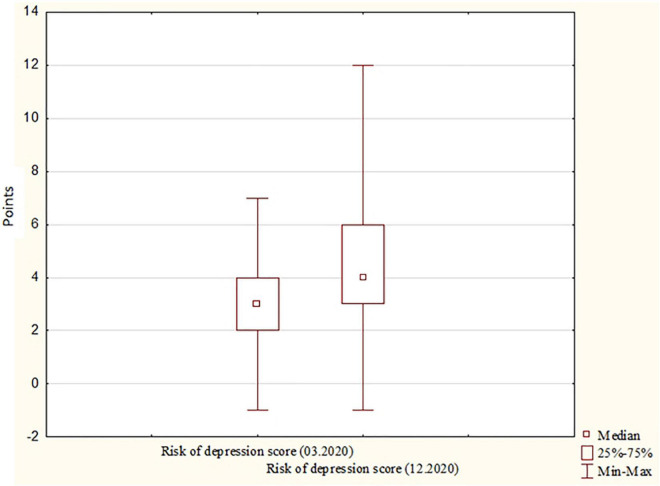
Risk of depression scores for March and December. Risk of depression scores, the number of scores obtained from the “Depressive Symptom Inventory.”

Significant differences (*p* < 0.0001) were also observed regarding the risk of depression: the median score recorded in March was lower than that recorded in December, which corresponded with a higher rate of moderate risk of depression in December compared to the first stage of the study. Risk of depression scores for March and December are presented in Figure 2.

In addition, the relationship between the degree of dementia and depression was analysed. The results obtained in December indicate a moderately strong (close to strong), negative correlation between the degree of dementia and the risk of depression: a lower the degree of cognitive impairment correlated with a higher the risk of depression (Table 2).

**TABLE 2:** Analysis of the relationship between the degree of dementia and the risk of depression in March and December.

Analysis of the relationship between the degree of dementia and the risk of depression in March (*p* = 0,7)		

Variables	MMSE interpretation* (03.2020)	Risk of depression** (03.2020)
MMSE interpretation (03.2020)	1,000	−0,023
Risk of depression (03.2020)	−0,023	1,000

**Analysis of the relationship between the degree of dementia and the risk of depression in December (*p* < 0,01)**		

**Variables**	MMSE interpretation* (12.2020)	Risk of depression** (12.2020)

MMSE interpretation (12.2020)	1,000	−0,492
Risk of depression (12.2020)	−0,492	1,000

*MMSE interpretation – MMSE scores interpreted according to the standard. ** Risk of depression – Depressive Symptom Inventory scores interpreted according to the scale.

With regard to the results obtained in December, the study group was characterised in terms of cognitive status in relation to the risk of depression (Table 3). A similar analysis was also performed for the results obtained in March, but the correlations were not statistically significant (*p* > 0.05). Based on the 12.2020 results, we found that impaired cognitive status (MMSE scores below 27 points) significantly affected the risk of depression – low risk of depression was most often noted among participants with severe dementia (*N* = 71; 50.0%), while high risk of depression was mainly characterised by subjects with normal cognitive status or cognitive impairment without dementia (MMSE scores of 30–27 points and 26–24 points, respectively). Respondents with mild and moderate dementia were most likely to have a medium risk of depression (29.0 and 27.4%, respectively). The differences between the groups were statistically significant.

**TABLE 3:** Cognitive status characteristics of the study group (MMSE interpretation) in relation to risk of depression (12.2020).

MMSE interpretation** (12.2020)	Risk of depression*** (12.2020)			*p*-value *
	Low	Moderate	High	
Normal score (30–27 points)	4; 2,8%	19; 15,3%	5; 71,4%	*p* < 0,01
Cognitive impairment without dementia (26–24 points)	7; 4,9%	21; 17,0%	2; 28,6%	
Mild dementia (23–19 points)	25; 17,6%	36; 29,0%	0; 0%	
Moderate dementia (18–11 points)	35; 24,7%	34; 27,4%	0; 0%	
Severe dementia (10–0 points)	71; 50,0%	14; 11,3%	0; 0%	

*Mann–Whitney U test. **MMSE interpretation – MMSE scores interpreted according to the standard. ***Risk of depression – Depressive Symptom Inventory scores interpreted according to the scale.

## Discussion

Healthcare professionals who work at various types of geriatric facilities encounter many problems of the elderly. One of the most serious difficulties, which is observed particularly during the pandemic, is separation from family and friends. This is reflected in an increase in the rate of depressive symptoms, diagnosis of depression and the use of antidepressants (Górski et al., 2020).

The present author’s observations indicate that at the beginning of the period of ban on visits in long-term care institutions, no differences were observed between residents with neurocognitive impairment and those without it with regard to symptoms of depression. However, as the social isolation continued, the differences became more noticeable. It was observed that certain characteristic symptoms of depression occurred in high-functioning individuals, able to communicate in a meaningful way and who had been frequently visited their families before the COVID-19 pandemic.

Cognitive impairment, regardless of the cause, is typically associated with compromised memory, particularly short-term memory, and problems with abstract thinking, connecting facts and understanding cause-and-effect relationships. Advanced cognitive impairment involves problems with deeper memory stores, including long-term and autobiographical memory. One’s life history, kinship relations and relationships with friends are forgotten (Han et al., 2017; Jia et al., 2020). Paradoxically, these alarming and adverse phenomena should be considered protective against depressive symptoms in the study group.

During the study, progression of cognitive impairment was observed in a relatively large group of subjects. A particular increase was noted in the moderate dementia category (from 18.7% in March to 25.3% in December) and severe dementia (from 28.2% in March to 31.1% in December); in addition, the number of subjects with a normal MMSE score decreased (by approximately 6%). Similar relationships were found in a study by Canevelli et al. (2020). In that study, in as many as 31.7% of respondents cognitive deterioration was observed during the period covered by the study, which is a higher result than in the present study. The observations mentioned above can be the result of a natural process associated with the progression of the underlying disease. It should be noted, however, that MMSE scores decreased relatively quickly and significantly, which may be due to isolation and the associated mental illness and trauma.

As demonstrated in the present study, the risk of depression was relatively higher in individuals with a normal MMSE score or cognitive impairment without dementia than in those with various degrees of dementia. The awareness of the pandemic and the associated danger has become particularly painful to elderly individuals, who are constantly thinking about the passing of time and the approaching end of life. This is particularly relevant to the notion of acceptance of one’s old age. Normal family relations which bring much satisfaction and a sense of security to the elderly individual are key to the acceptance of slow decline in body strength, activity, loss of health and ultimately death. Unfortunately, due to the development of the pandemic, contacts with loved ones were restricted, which resulted in intensified pessimistic attitude, reluctance to leave one’s room, increased weepiness and catastrophic thinking.

The influence of the media also takes its toll. The media are an important information channel for the local, national and international community. Currently, the media reports are dominated by news on the pandemic, daily incidence rates, complications and deaths (Bilal et al., 2020; Canevelli et al., 2020). There were also reports on shortage of staff, free beds in hospitals and necessary equipment (e.g., ventilators). All of this undoubtedly had an effect on the mood of individuals from various social and age groups, particularly the elderly, who are a high-risk group due to their age. One may suspect that such information could have intensified pessimistic thinking or exacerbated anxiety disorders in the study group. Such a supposition is highly likely for individuals with no cognitive impairment who have free access to the media. Individuals with dementia are in a different situation. They have limited access to media reports and even if they do encounter some news reports, they may not understand them due to the lack of their placement in appropriate context. On the one hand, this leads to the risk of lack of information on appropriate safety measures (wearing face masks, frequent hand washing, sanitation), and on the other hand, it reduces the associated high stress (Wang et al., 2020). Similar conclusions were drawn from other studies. Bilal et al. (2020) indicated that the news policy on COVID-19 causes panic and depression. Furthermore, Yan et al. (2020) believe that the media one of the main factors affecting mental health during the COVID-19 pandemic. However, some credit should also be given to the media in the fight against the COVID-19 pandemic. As demonstrated by Zhou et al. (2020) broadcasting information on ways of infection prevention contributed to an increase in social awareness of the problem, reduced the peak of infection rates and decreased transmission at the initial stage of the pandemic.

Clinical observation of patients resulted in identifying certain characteristic features of the subjects. In elderly individuals, reluctance to leave their room, decline in general life activity, frequent inquiries about their family, sadness and concern over their health were most frequently observed. In individuals with cognitive impairment, the main observations included general anxiety and decline in independence, defined as helplessness and impaired execution of daily activities such as washing, getting dressed and eating. These data are corroborated in the literature (Brown et al., 2020; Canevelli et al., 2020). Brown et al. (2020) report that during the isolation associated with COVID-19, behavioural disorders, such as increased anxiety, psychomotor agitation, wandering and increased orientation disturbances are observed, which may result in the cessation of purposeful activity. Similar findings were presented in the study by Rainero et al. (2021), which is the largest study on the relationship between pandemic COVID-19 and dementia patients. It proved that cognitive and behavioural deterioration was observed in more than half of the patients. It was noted that quarantine exposure was associated with an acute worsening of clinical symptoms in patients with dementia, as well as an increased burden on caregivers (Rainero et al., 2021). It is also important to note the interrelationship between cognitive function decline and depression. Yoon et al. (2017) demonstrated that patients diagnosed with depression showed worse cognitive functioning than patients without depression. Moreover, the introduction of depression treatment was associated with improvement or prevention of cognitive decline. Similar relationships were noted in the study by Anderson et al. (2020). It proved that when depression was remitted, patients’ functioning improved in some components of cognitive functioning, e.g., self-memory, autobiographical memory or category verbal fluency.

However, special attention should be paid to the study by Donovan et al. (2017). The cited study examined 8382 respondents over the age of 65 years. It evaluated the mutual influence of factors such as, loneliness, cognitive functioning and depression. The authors demonstrated that loneliness significantly influenced cognitive decline regardless of socio-demographic factors, social network, poor health and baseline depression. Our own results appear to support these findings. Social isolation caused by the development of the COVID-19 pandemic increased the prevalence of depressive symptoms and exacerbated cognitive deficits. The reciprocal effect of depression on cognitive functioning and, conversely, of cognitive functioning on depression remains undisputed, but we suspect that in our study, as in the Donovan study (Donovan et al., 2017), social isolation and loneliness are the main factors affecting both.

The impact of the COVID-19 pandemic has been vigorously discussed in the Polish and international community of psychologists, psychiatrists, geriatric psychologists, geriatricians and gerontologists. Clinicians notice certain differences in the functioning of individuals with and without cognitive impairment during the pandemic; however, to date, no study has been found that would demonstrate the existence of such differences in a scientific manner. This study is intended to fill this gap at least to a small extent. This is not a perfect study and we are aware of certain limitations. The drawbacks of this study is the fact that no standardised questionnaire has been used. The choice of a non-standardised questionnaire was based on the fact that none of the widely available standardised questionnaires makes it possible to conduct such a study in individuals with cognitive impairment. Tests such as the Geriatric Depression Scale (GDS) or Beck Depression Inventory (BDI), require self-awareness and appropriate critical approach to one’s feeling, behaviour, attitudes and health status, which are lost in individuals affected by cognitive impairment. Moreover, depression in elderly individuals are often masked with other symptoms than sadness; for this reason, particular emphasis was placed in the study on behaviour that is possible to asses by the staff, not just emotions and feelings of the patient (Guimarães et al., 2019; El Haj et al., 2020). The study group was a population of patients residing at long-term care institutions in, which made it impossible to select the group in terms of the number of patients with and without depression according to the duration of the pandemic. The lack of statistical significance in the first study period (March) may be related to the too small group of residents with depression. This fact should be considered a limitation of the study. A simplified correlation between MMSE scores and “Depressive Symptom Inventory” was used in this research. Selected confounding criteria such as, education level, comorbidities, functional or physical activity level were not considered, which requires additional analyses in the future and is a limitation of this study.

In conclusion, a higher risk of depression was observed with the development of the pandemic. At the initial stage of the COVID-19 pandemic (March) the risk was assessed as low and in the second stage of the study (December), the risk significantly increased. Residents with cognitive impairment are characterised by a lower risk of depression compared to individuals with a normal MMSE score. It is believed that the reason for this is prolonged period of isolation, which had an adverse effect on the mental state of the participants who had a high level of functioning before the COVID-19 pandemic. The pandemic also affected MMSE scores. In the course of the study, progression of cognitive impairment was observed in the residents, apart form that, the proportion of subjects with a normal MMSE score decreased. What is more, the results of the present study show that the awareness of danger plays and important role in coping with the COVID-19 pandemic. Factors such as isolation, sense of lack of security, stress, pessimistic thoughts and general decrease in mood may result in the development of depression and cognitive impairment.

## Data Availability Statement

The original contributions presented in the study are included in the article/supplementary material, further inquiries can be directed to the corresponding author/s.

## Author Contributions

MGó led the conception of work and analyses, as well as the interpretation of data for publication and the writing of the manuscript. JG, MGr, BC, KP, and AG contributed to the design and the writing of the manuscript, namely preparation and critical revision. MB supervised the analyses. RP was the primary reviewer of the manuscript and contributed many of the final revisions. All authors have read and approved the final version for publication and contributed to the article and approved the submitted version.

## Conflict of Interest

The authors declare that the research was conducted in the absence of any commercial or financial relationships that could be construed as a potential conflict of interest.

## Publisher’s Note

All claims expressed in this article are solely those of the authors and do not necessarily represent those of their affiliated organizations, or those of the publisher, the editors and the reviewers. Any product that may be evaluated in this article, or claim that may be made by its manufacturer, is not guaranteed or endorsed by the publisher.

## Abbreviations

BDI: beck depression inventory
CCDC: chinese center for disease control and prevention
COVID-19: coronavirus disease
CT scan: computed tomography scan
GDS: geriatric depression scale
HIV: the human immunodeficiency viruses
MMSE: mini mental state examination
MRI: magnetic resonance imaging
SARS-CoV-2: severe acute respiratory syndrome coronavirus 2
WHO: world health organization.
